# A B7-CD28 Family-Based Signature Demonstrates Significantly Different Prognosis and Immunological Characteristics in Diffuse Gliomas

**DOI:** 10.3389/fmolb.2022.849723

**Published:** 2022-07-19

**Authors:** Xiangyang Deng, Kezhu Chen, Junwei Ren, Jun Zeng, Quan Zhang, Tianwen Li, Qisheng Tang, Jianhong Zhu

**Affiliations:** Fudan University Huashan Hospital, Department of Neurosurgery, National Center for Neurological Disorders, National Key Laboratory for Medical Neurobiology, Shanghai Key Laboratory of Brain Function and Regeneration, MOE Frontiers Center for Brain Science, Institutes of Brain Science, Shanghai Medical College-Fudan University, Shanghai, China

**Keywords:** B7, CD28, prognostic signature, diffuse glioma, TCGA, CGGA

## Abstract

The B7-CD28 gene family plays a crucial role in modulating immune functions and has served as potential targets for immunotherapeutic strategies. Therefore, we systematically analyzed B7-CD28 family gene expression profiles and constructed a B7-CD28 family-based prognostic signature to predict survival and immune host status in diffuse gliomas. The TCGA dataset was used as a training cohort, and three CGGA datasets (mRNAseq_325, mRNAseq_693 and mRNA-array) were employed as validation cohorts to intensify the findings that we have revealed in TCGA dataset. Ultimately, we developed a B7-CD28 family-based signature that consisted of CD276, CD274, PDCD1LG2 and CD80 using LASSO Cox analysis. This gene signature was validated to have significant prognostic value, and could be used as a biomarker to distinguish pathological grade and IDH mutation status in diffuse glioma. Additionally, we found that the gene signature was significantly related to intensity of immune response and immune cell population, as well as several other important immune checkpoint genes, holding a great potential to be a predictive immune marker for immunotherapy and tumor microenvironment. Finally, a B7-CD28 family-based nomogram was established to predict patient life expectancy contributing to facilitate personalizing therapy for tumor sufferers. In summary, this is the first mathematical model based on this gene family with the aim of providing novel insights into immunotherapy for diffuse glioma.

## Introduction

Diffuse glioma, including low-grade glioma (LGG) and glioblastoma (GBM), is the most common prevalent and devastating primary tumor in central nervous system, accounting for approximately 80% of malignant brain tumors (Weller et al., 2015; Aquilanti et al., 2018; Hu et al., 2019). Despite the remarkable progress has been made in neurosurgical resection, radiotherapy, adjuvant chemotherapy and targeted therapy, the clinical efficiency and prognosis of glioma patients have not improved significantly. And GBM is still one of the hardest cancers to treat in clinical oncology, with an overall 5-years survival rate of only 9.8% (Li et al., 2017; Wang et al., 2018). Thus, intensive study of these tumors should be conducted to discover specific biomarkers for prognosis prediction and clinical management optimization. Although amounts of researchers have taken advantage of genes from the whole genome or transcriptome modeling to predict glioma outcomes, for their little consideration of biological function of selected genes, most of these signatures were simply mathematic models without the ability to reflect the innate character of cancer, and had a limited role.

Immune checkpoint inhibitors targeting the B7-CD28 family members have shown clinically relevant efficacy in a number of tumor types and have revolutionized the strategy in cancer treatment (Mahoney et al., 2015; Picarda et al., 2016; Ramagopal et al., 2017). B7 ligands are widely expressed on the membrane of antigen-presenting cells, while CD28 receptors are widely expressed on T cells; and the interplay between these molecules play critical and unique roles in T-cell co-stimulation and co-inhibition (Chen and Flies, 2013; Schildberg et al., 2016). Manipulation of the interactions between B7 ligands and CD28 receptors holds great potential to enhance anti-tumor immunity and has emerged as a novel treatment paradigm. Moreover, efficacy of programmed cell death 1/programmed death ligand 1(PD1/PD-L1) inhibitors has been reported in preclinical glioma models and in individual human cases (Reardon et al., 2016; Berghoff et al., 2017; Kim et al., 2017). Thus, continued efforts to explore clinical and prognostic value of B7-CD28 family members are warranted.

Some investigations have focused on several single B7-CD28 family members, nevertheless, comprehensive understanding of B7-CD28 family members is still needed to decode complex interaction between tumor and immunity. In this article, we systematically analyzed the prognostic value of the B7-CD28 family in diffuse glioma and construct a B7-CD28 family-based prognostic signature. We further investigated the correlation of the signature with clinicopathologic, molecular, and immunological characteristics of the tumor, which might provide novel insights into the glioma immune microenvironment and immunotherapy. Furthermore, a B7-CD28 family-based predictive nomogram model was developed to estimate survival for glioma patients.

## Materials and Methods

### Data Collection

In this article, four datasets were obtained for the analysis. In the TCGA-GBMLGG dataset, RNA‐Seq data were extracted from UCSC Xena database, and the clinical data and survival information were downloaded from https://tcga-data.nci.nih.gov/docs/publications/lgggbm_2016/. Then, three CGGA datasets (mRNAseq_325, mRNAseq_693 and mRNA-array) containing gliomas of all grades were employed as validation cohorts to intensify the findings that we have revealed in TCGA dataset. And the corresponding clinical pathological parameters were also download form the CGGA database. The transcriptome profiling of RNA measured by FPKM values was performed using the log2-based transformation for further analysis. After excluding patients with unknown survival information or survival time of 0, a total of 1829 patients were identified for further analysis. The clinical information of patients in each dataset is summarized in Table 1.

**TABLE 1:** Clinical characteristics of the patients.

Characteristics	TCGA	mRNAseq_325	mRNAseq_693	mRNA-array
Total	604 (100%)	310 (100%)	617 (100%)	298 (100%)
Age, y				
≤47	308 (51.0%)	205 (66.1%)	407 (65.9%)	198 (66.4%)
>47	296 (49.0%)	105 (33.9%)	210 (34.0%)	98 (32.9%)
NA			1 (0.2%)	2 (0.7%)
Sex				
Male	350 (57.9%)	193 (62.3%)	356 (57.6%)	177 (59.4%)
Female	254 (42.1%)	117 (37.7%)	262 (42.4%)	121 (40.6%)
Grade				
Grade II	213 (35.3%)	97 (31.3%)	173 (28.0%)	115 (38.6%)
Grade III	238 (39.4%)	74 (23.9%)	231 (37.4%)	57 (19.1%)
Grade IV	153 (25.3%)	135 (43.5%)	214 (34.6%)	123 (41.3%)
NA		4 (1.3%)		3 (1%)
IDH status				
Wildtype	224 (37.1%)	142 (45.8%)	258 (41.7%)	164 (55.0%)
Mutant	374 (61.9%)	168 (54.2%)	315 (51.0%)	132 (44.3%)
NA	6 (1%)		45 (7.3%)	2 (0.7%)
MGMT promoter status				
Unmethylated	148 (24.5%)			
Methylated	425 (70.4%)			
NA	31 (5.1%)			
TERT promoter status				
Wildtype	160 (26.5%)			
Mutant	154 (25.5%)			
NA	290 (48.0%)			
Radiotherapy				
No	172 (28.5%)	46 (14.8%)	102 (16.5%)	37 (12.4%)
Yes	374 (61.9%)	253 (81.6%)	485 (78.5%)	249 (83.6%)
NA	58 (9.6%)	11 (3.5%)	31 (5.0%)	12 (4.0%)
Chemotherapy				
No		120 (38.7%)	139 (22.5%)	126 (42.3%)
Yes		175 (56.5%)	435 (70.4%)	151 (50.7%)
NA		15 (4.8%)	44 (7.1%)	21 (7.0%)

Abbreviations: NA, not available.

### Statistical Analysis and Bioinformatic Analysis

Univariate Cox regression analysis was conducted to access the association between the expression of each B7-CD28 family gene and overall survival. Then, the least absolute shrinkage and selection operator (LASSO) method was employed to identify the genes with best prognostic value and establish a risk score equation (Tibshirani, 1997; Goeman, 2009). Patients with assigned risk scores then were separated into high- and low-risk groups using the median risk score as the cutoff point. Kaplan-Meier method with log-rank test was utilized to compare survival differences between different groups. Multivariate Cox regression analysis was performed to evaluate the independent prognostic value of the B7-CD28 family-based signature.

The Estimation of STromal and Immune cells in Malignant Tumours using Expression data (ESTIMATE) algorithm was employed to calculate the immune and stromal scores (Yoshihara et al., 2013). The Microenvironment Cell Populations-counter (MCP) method was utilized to evaluate the relationship between the gene signature and tumor microenvironment (Becht et al., 2016). After Spearman correlation analysis, gene set enrichment analysis (GSEA) analysis was performed to explore biological functions associated with the gene signature (Subramanian et al., 2005). Gene sets used in this work were downloaded from the Molecular Signatures Database (http://software. broadinstitute.org/gsea/msigdb/index.jsp). Gene Sets Variation Analysis (GSVA) was also used to access the inflammatory activities in glioma microenvironment, as previously described (Rody et al., 2009; Hänzelmann et al., 2013).

To individualize the 1-, 3- and 5-years predicted overall survival probability, a nomogram was constructed based on the results of the multivariate analysis. Calibration curves were depicted to access the consistency between nomogram-predicted survival and actual outcome. Discrimination ability of the nomogram was evaluated by concordance index (C-index), and time-dependent receiver operating characteristic curve (ROC) with the area under the curve (AUC) value (Heagerty et al., 2000). All statistical analyses were conducted using R project (version 3.5.2, https://www.r-project.org/). A two-sided *p* value <0.05 was regarded as significant.

## Results

### Establishment of the B7-CD28 Family-Based Signature and Evaluation of its Prognostic Value in TCGA Cohort

**FIGURE 1:**
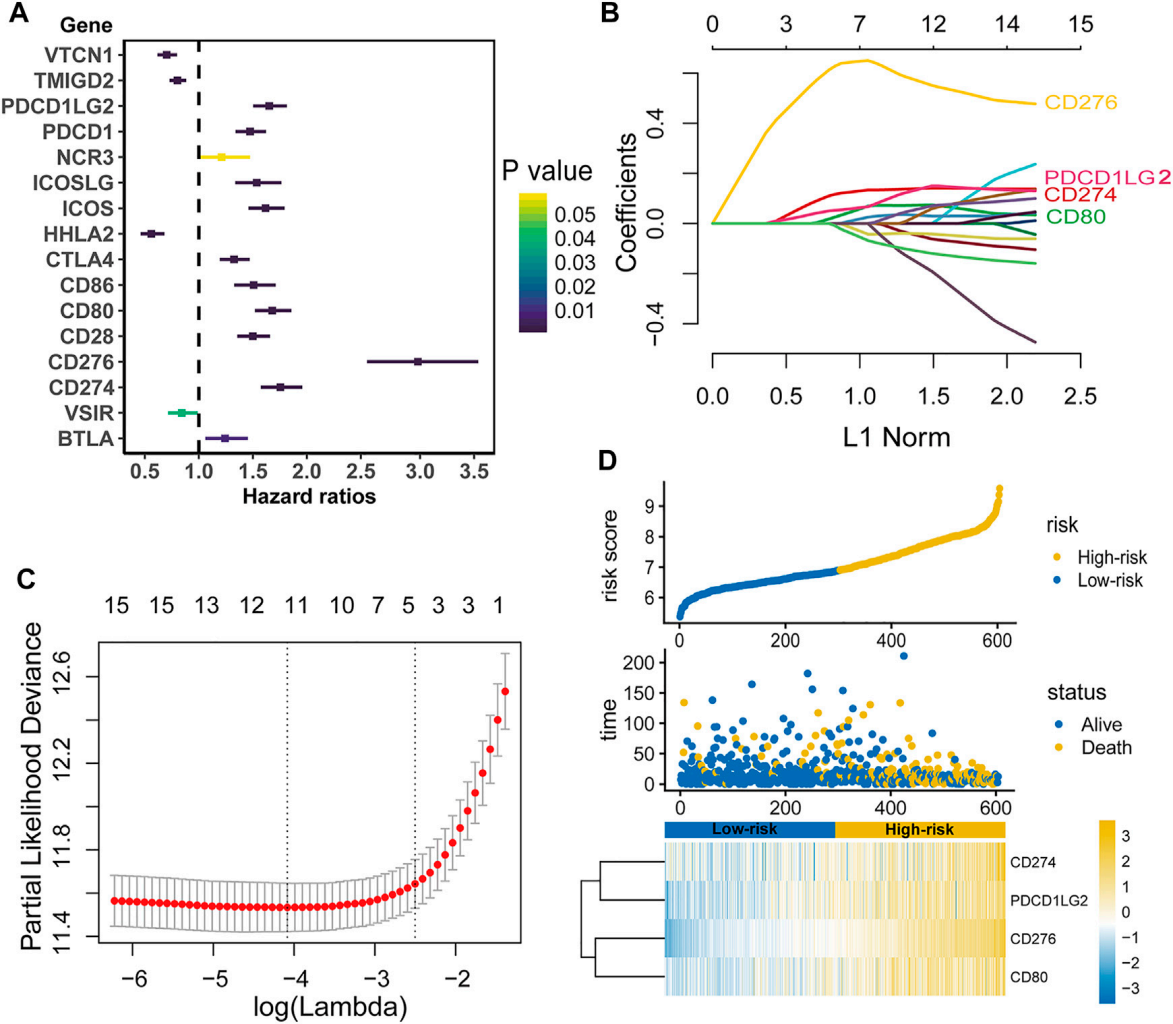
Establishment of the B7-CD28 family-based signature. Individual B7-CD28 gene univariate Cox analysis in TCGA Cohort **(A)**. LASSO Cox analysis identified four genes most correlated to overall survival in TCGA cohort **(B and C)**. Risk scores distribution, survival status of each patient, and the heatmap of B7-CD28 family-based signature in TCGA cohort **(D)**.

**FIGURE 2:**
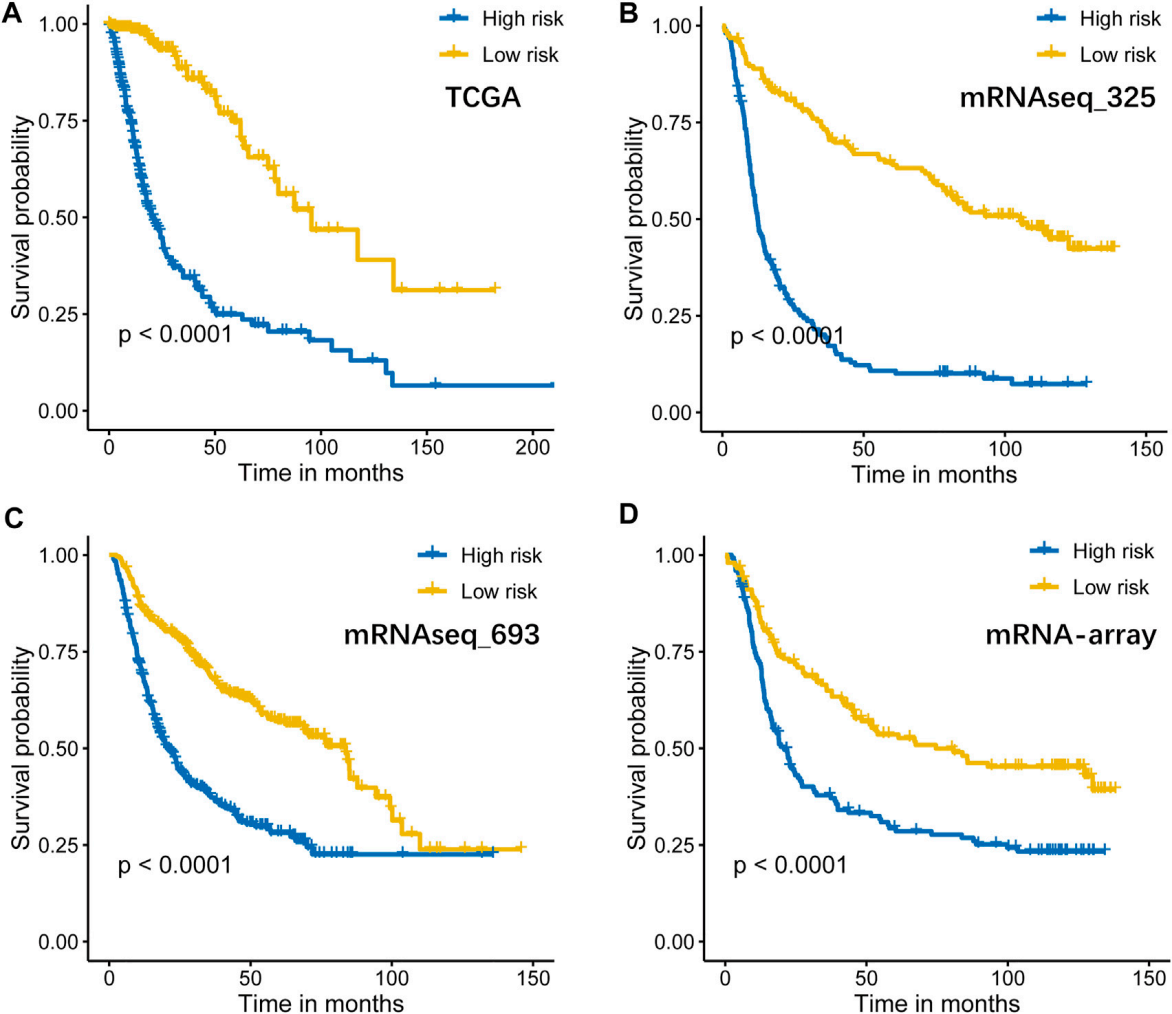
Kaplan–Meier curves of overall survival for high-risk and low-risk TCGA (*n* = 604, **(A)**], mRNAseq_325 (*n* = 310, **(B)**], mRNAseq_693 (*n* = 617, **(C)**], and mRNA-array (*n* = 298, **(D)**] patients.

The TCGA dataset was used as a training cohort, and we investigated the expression of sixteen well defined B7-CD28 family genes in the 604 diffuse glioma patients from this cohort. As shown in Figure 1A, almost all genes were significantly associated with prognosis in univariate Cox regression analysis, suggesting that the important role of this gene family in glioma outcome. Specifically, increased expression of eleven genes (PDCD1LG2, PDCD1, ICOSLG, ICOS, CTLA4, CD86, CD80, CD28, CD276, CD274 and BTLA) were significantly associated with worse survival, while the other four genes (VTCN1, TMIGD2, HHLA2 and VSIR) were related to better outcomes. Then, LASSO Cox analysis was performed to select best prognostic features and build the gene signature. Ultimately, a B7-CD28 family-based signature was developed using CD276, CD274, PDCD1LG2 and CD80 (Figures 1B,C). Risk scores were calculated for each patient (risk score = 0.613*CD276 + 0.109*CD274 + 0.050*PDCD1LG2 + 0.018*CD80). And patients were assigned into high- and low-risk groups by their risk scores using the median risk score as cutoff point (Figure 1D). In Kaplan-Meier analysis, the high-risk patients had shorter survival times than their low-risk counterparts (*p* < 0.05; Figure 2A). After adjusting for available clinicopathological variables, multivariate Cox analysis revealed that the prognostic signature-based risk score remained an independent prognostic factor (HR 2.581, 95%CI 1.503–4.434, *p* = 0.001, Table 2).

**TABLE 2:** Multivariate Cox regression analysis of the B7-CD28 family-based signature and characteristics with.

Characteristics	TCGA		mRNAseq_325		mRNAseq_693		mRNA-array	
	HR (95%CI)	P	HR (95%CI)	P	HR (95%CI)	P	HR (95%CI)	P
Risk score								
High vs. Low	2.581 (1.503–4.434)	0.001	2.826 (1.943–4.108)	<0.001	1.555 (1.209–2.001)	0.001	1.362 (0.988–1.879)	0.060
Age								
>47 vs. ≤47	2.661 (1.666–4.252)	<0.001	1.574 (1.110–2.234)	<0.001	1.144 (0.898–1.459)	0.277	1.642 (1.175–2.293)	0.004
Sex								
Female vs. Male	0.939 (0.672–1.312)	0.711	1.117 (0.825–1.511)	0.475	0.939 (0.749–1.175)	0.581	0.906 (0.666–1.231)	0.528
Grade								
Grade IV vs. Grade II/III	2.523 (1.561–4.076)	<0.001	2.633 (1.835–3.778)	<0.001	2.580 (1.924–3.459)	<0.001	3.501 (2.382–5.146)	<0.001
IDH status								
Mutant vs. Wildtype	0.281 (0.156–0.506)	<0.001	1.118 (0.765–1.634)	0.556	0.509 (0.384–0.674)	<0.001	0.878 (0.598–1.291)	0.508
MGMT promoter status								
Methylated vs. Unmethylated	0.882 (0.594–1.308)	0.531						
TERT promoter status								
Mutant vs. Wildtype	0.860 (0.490–1.497)	0.594						
Radiotherapy								
Yes vs. No	0.485 (0.312–0.754)	<0.001	0.673 (0.466–0.971)	0.034	1.023 (0.711–1.471)	0.903	0.519 (0.324–0.830)	0.006
Chemotherapy								
Yes vs. No			0.858 (0.627–1.173)	0.337	0.959 (0.693–1.328)	0.758	1.078 (0.775–1.499)	0.655

Overall survival.

Abbreviations: HR, hazard ratio; CI, confidence intervals.

### Validation of the B7-CD28 Family Gene-Based Signature in CGGA Cohorts

To determine whether the B7-CD28 four-gene signature has robust prognostic value, the performance of the signature was also assessed in other three cohorts from CGGA databases. Consistent with the results of the TCGA cohort, patients who were divided into high-risk group had significantly worse outcomes than those in low-risk group (All *p* < 0.05; Figures 2B–D). Multivariate Cox analysis indicated that risk score a constantly independent role for predicting glioma survival, although with a borderline significance in CGGA mRNA-array dataset (Table 2).

### Validation of the B7-CD28 Four Gene-Based Signature in Clinically Important Subsets.

**FIGURE 3:**
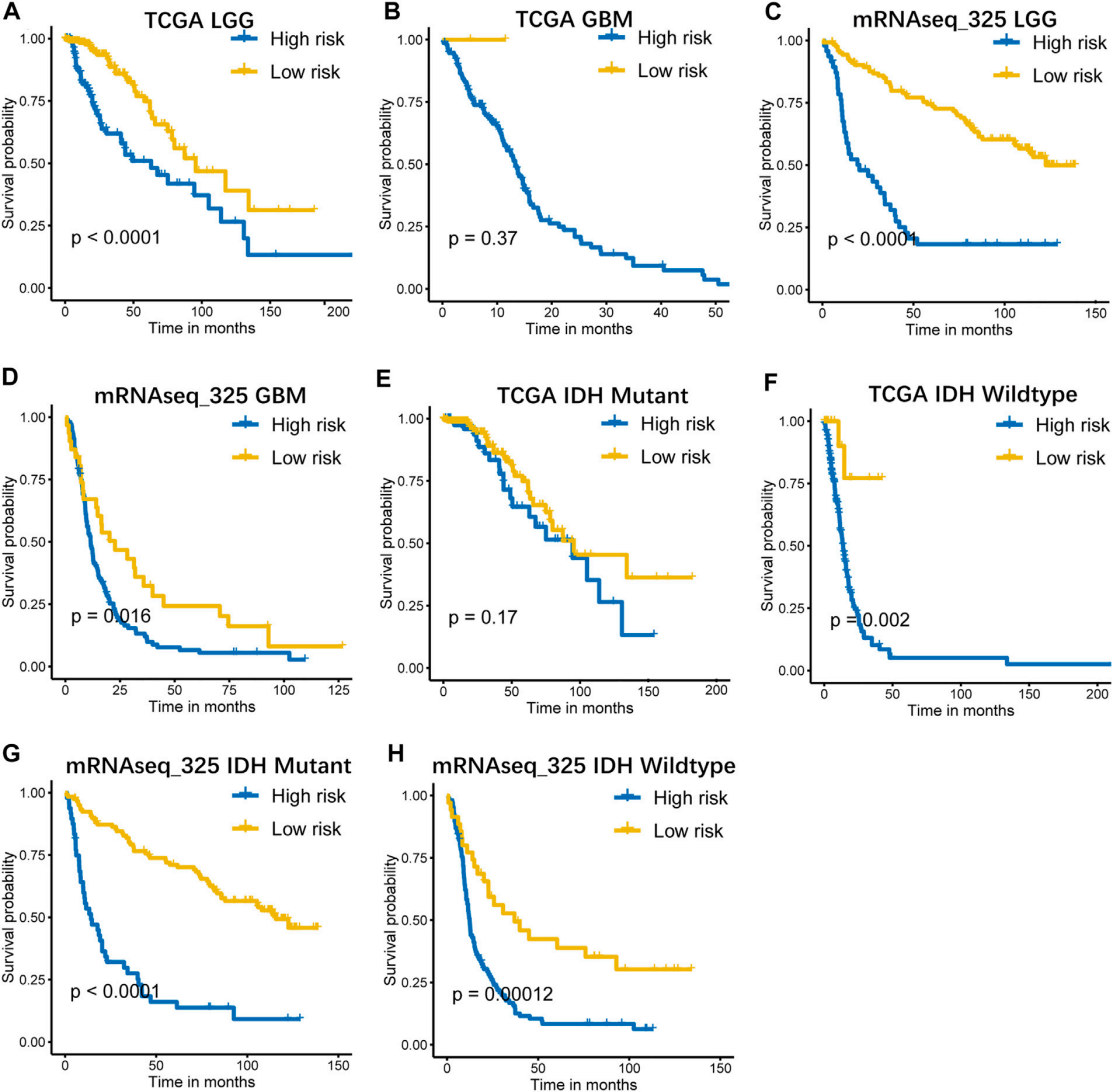
Kaplan–Meier curves of overall survival for high-risk and low-risk patients stratified by pathological grade **(A–D)**, IDH status **(E–H)** in TCGA and mRNAseq_325 cohorts. LGG, low grade glioma; GBM, glioblastoma.

Considering the histopathological heterogeneity of the glioma, the prognostic value of the signature was further analyzed according to WHO grade system and IDH mutation status. Stratification analyses were carried out and showed that our signature accurately predicted survival in LGG patients. However, its predictive accuracy seemed to be relatively poor in GBM patients which may due to the small number of low-risk cases in GBM subgroups (Figures 3A–D and Supplementary Figure S1A–D). In addition, the performance of the signature was observed to be favorable in patient subsets with IDH wildtype, whereas it did not work quite as well in IDH mutant subgroups (Figures 3E–H and Supplementary Figure S1E–H).

### Correlation of the Prognostic Signature With Pathological and Molecular Characteristics in Glioma

**FIGURE 4:**
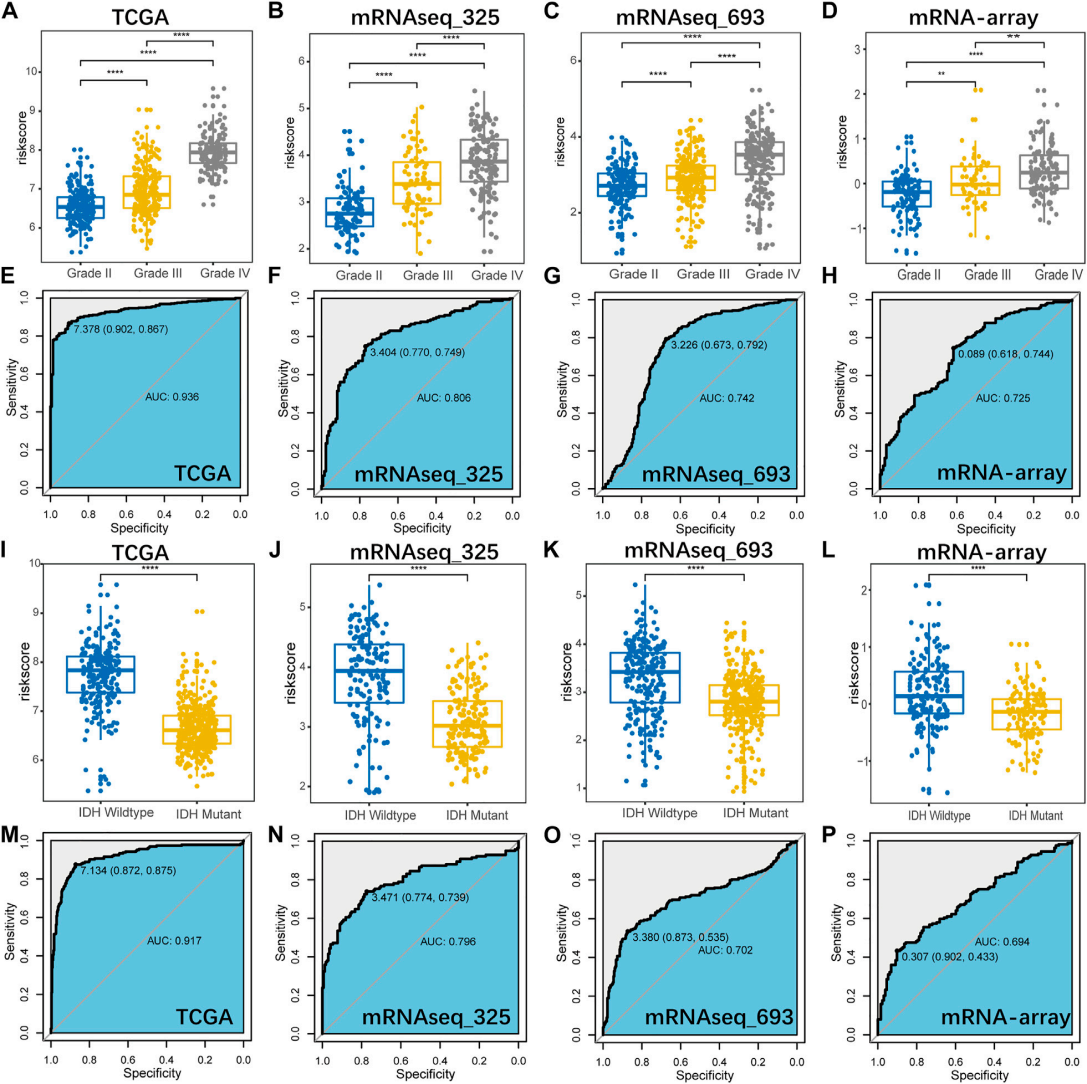
The distribution of the signature-based risk scores in stratified patients by pathological grade **(A–D)** and IDH status **(I–L)** in all cohorts. ROC curve analysis showed that B7-CD28 family-based signature had highly sensitivity and specificity to predict pathological grade **(E–H)** and IDH status **(M–P)** in diffuse glioma. ∗∗, ∗∗∗ and ∗∗∗∗ represent *p* < 0.01, *p* < 0.001 and *p* < 0.0001, respectively.

Then, we further investigated the distribution of the risk score on basis of the WHO grade and IDH mutation status, to explore the correlation of the prognostic signature with these parameters in glioma. And ROC curve analysis was also performed to evaluate its predictive value. As illustrated in Figures 4A–D, I–L, we found that risk score was significantly higher in GBM (Grade IV vs. Grade II/III) and IDH wildtype (IDH wildtype vs. IDH mutant) subgroups. ROC curve analysis also suggested that this gene signature could serve as a biomarker to distinguish pathological grade and IDH mutation status in diffuse glioma (Figures 4E–H, M–P).

### Correlation of the Prognostic Signature With the Tumor Microenvironment

**FIGURE 5:**
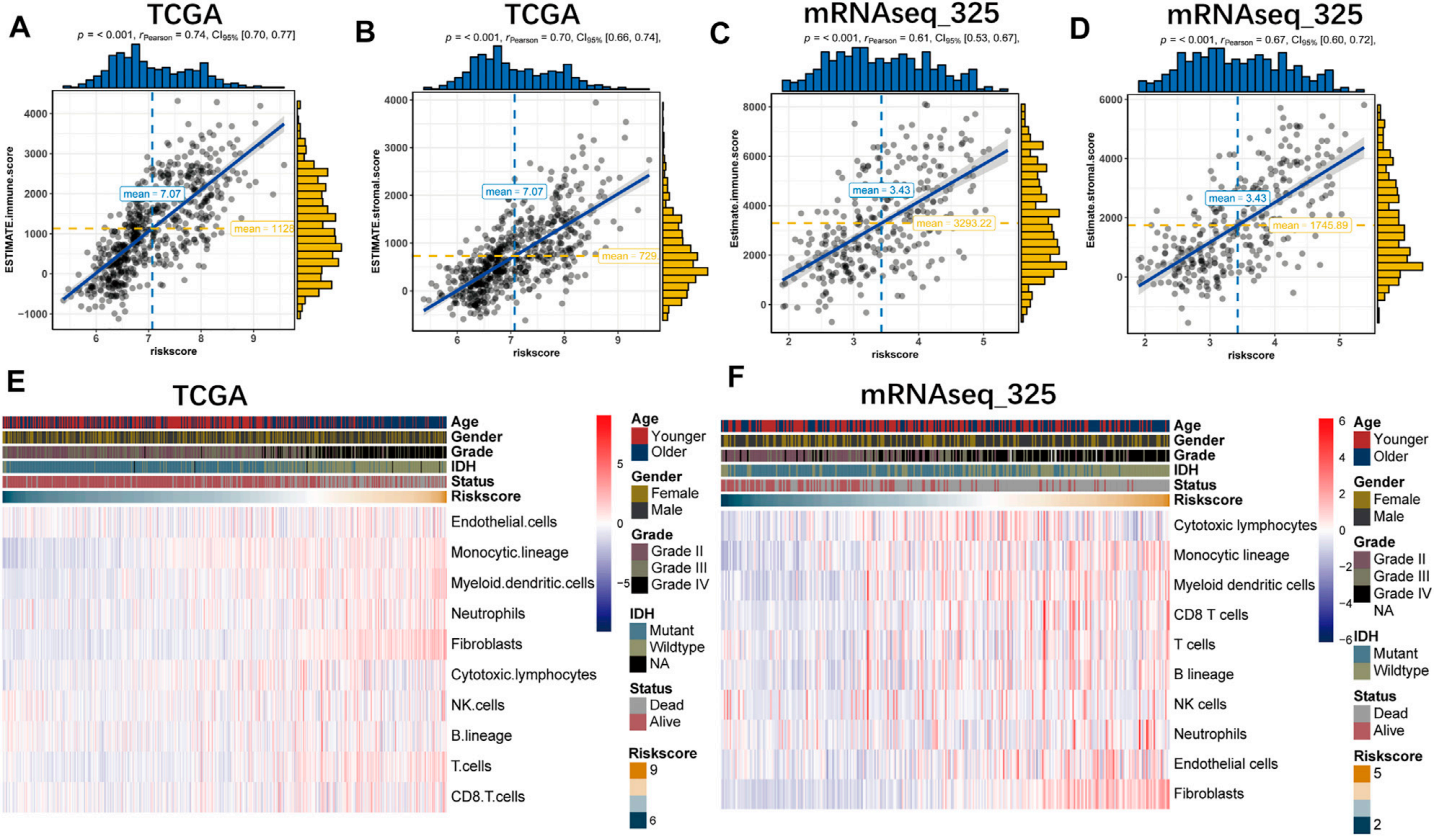
The B7-CD28 family-based signature was tightly associated with immune score **(A,B)**, stromal score **(C,D)** and infiltrated cells in tumor microenvironment **(E,F)** in TCGA and mRNAseq_325 cohorts.

ESTIMATE and MCP analyses were conducted to investigate the relationship between the prognostic signature and glioma microenvironment. And we found that the risk score was positively related to the immune and stromal scores in all datasets based on ESTIMATE algorithm (Figure 5A–D and Supplementary Figure S2A–D). With the MCP method, we further explored the association of the gene signature with specific cell populations in the tumor microenvironment. The findings revealed that risk score was significantly associated with immune cell population, especially with myeloid dendritic cells, monocytic lineage and fibroblasts (Figure 5E,F and Supplementary Figure S2E,F).

### The Prognostic Signature-Related Biological Process

**FIGURE 6:**
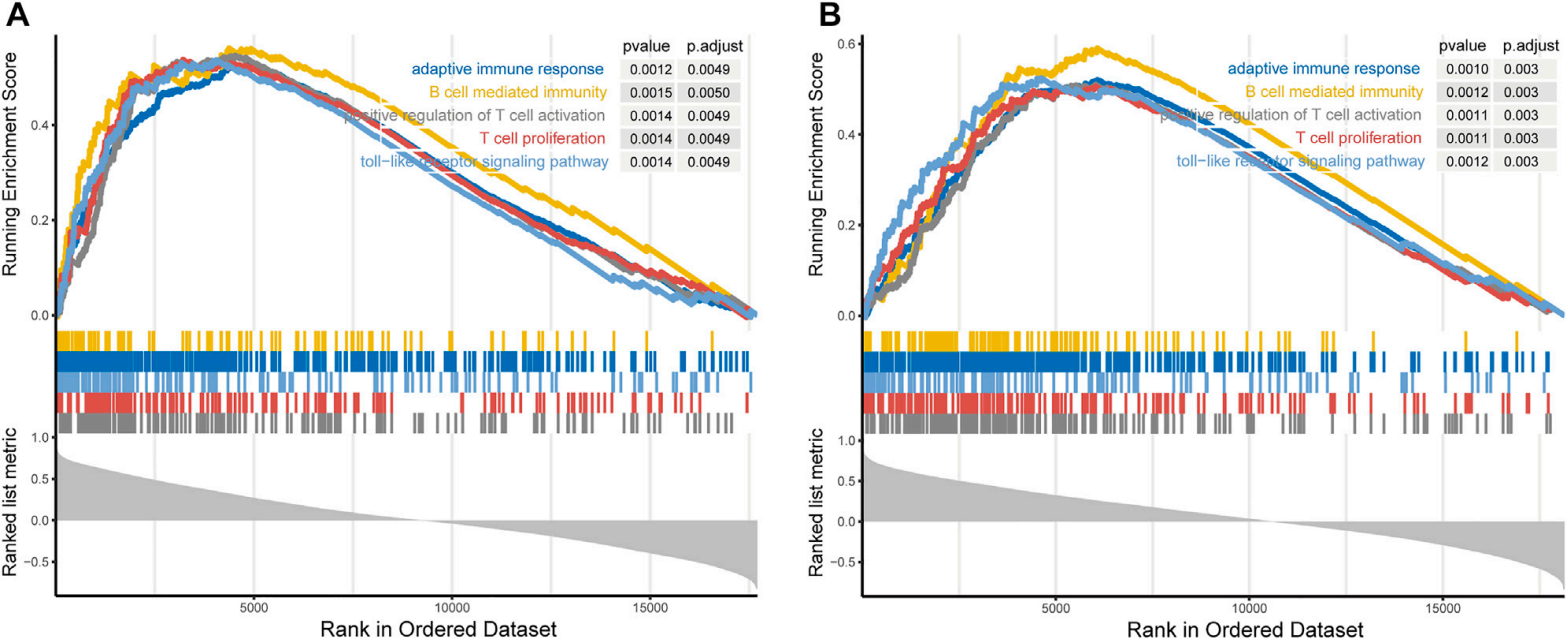
Gene set enrichment analysis indicated that genes positively correlated with the signature-based risk score were mainly enriched in adaptive immune response, B cell mediated immunity, positive regulation of T cell activation, T cell proliferation and toll-like receptor signaling pathway in TCGA **(A)** and mRNAseq_325 cohorts **(B)**.

Since the prognostic signature was significantly related to tumor malignancy and microenvironment, GSEA analysis was performed to explore the potential biologic functions. First, we calculated the correlation between risk score and all genes. After GSEA analysis, we found that genes positively correlated with risk score (ranked by Spearman lRl) were mainly enriched in adaptive immune response, B cell mediated immunity, positive regulation of T cell activation, T cell proliferation and toll-like receptor signaling pathway (Figure 6A,B and Supplementary Figure S3A,B).

### Correlation of the Prognostic Signature With Inflammatory Response

**FIGURE 7:**
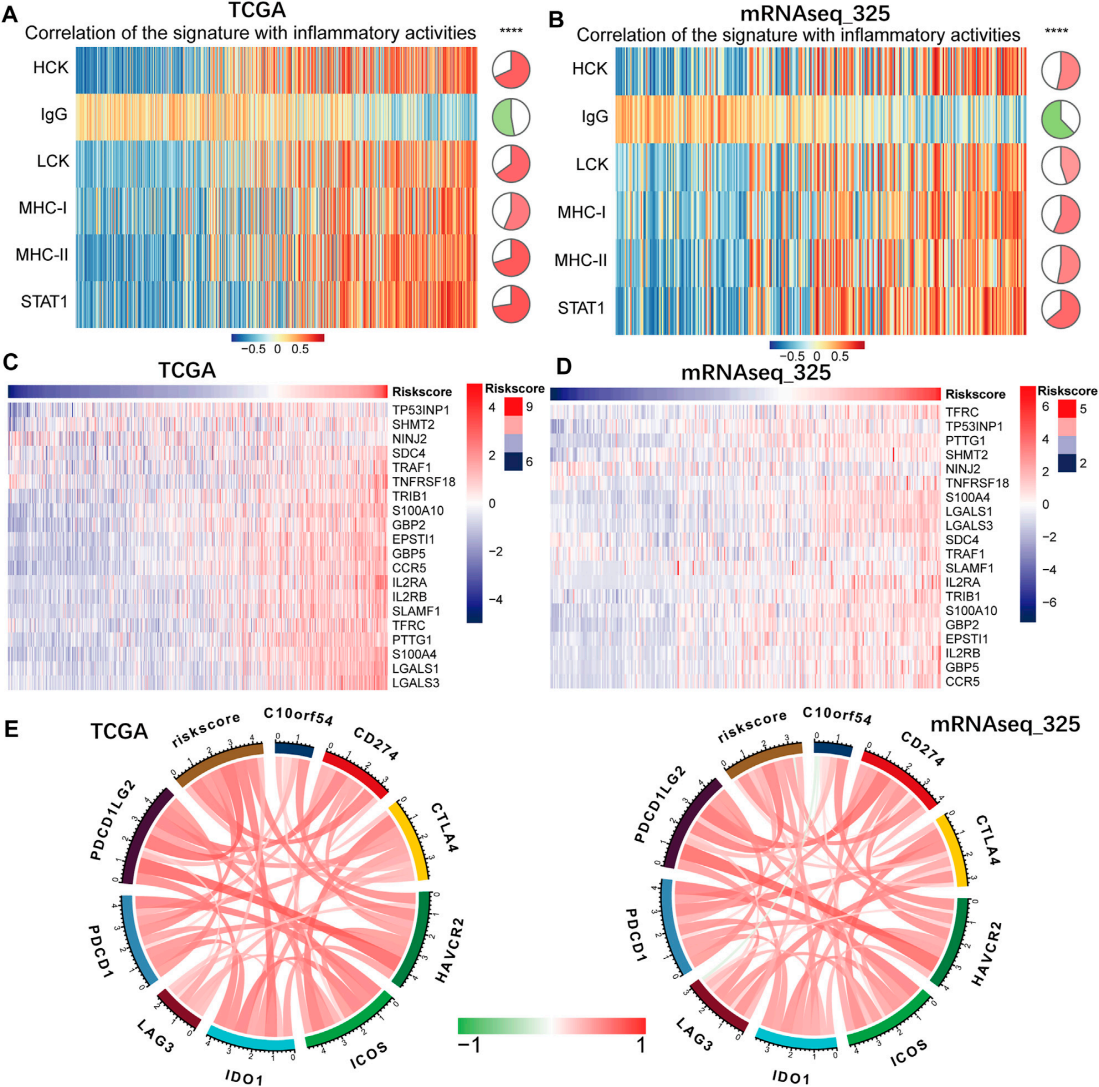
The associations of the B7-CD28 family-based signature with inflammatory activities **(A,B)**, Treg signatures expression **(C,D)** and several checkpoint members **(E)**. ∗∗∗∗ represents *p* < 0.0001 in TCGA and mRNAseq_325 cohorts.

Given the strong association of the gene signature with immunologic biological processes, we further performed GSVA analysis with six inflammatory metagene clusters to specifically analyze the relationship between the gene signature and inflammatory response (Supplementary Table S1). The results showed that the signature-based risk score was positively related to HCK, LCK, MHC-I, MHC-II and STAT1, but negatively associated with IgG (Figure 7A,B and Supplementary Figure S4A,B). As a subpopulation of T cells, the regulatory T cells (Tregs), formerly known as suppressor T cells, play an important role in regulation of the immune system (Severin et al., 2016). Then we attempted to investigate the association between the risk score and Treg signatures (Supplementary Table S2). And we found that risk score was significantly positively related to Treg signatures expression (Figures 7C,D and Supplementary Figure S4C,D). In sum, these results demonstrated that B7-CD28 four gene-based signature was related to inflammation cells transduction signals activation and immunosuppressive functions.

### Correlation of the Prognostic Signature With Immune Checkpoints

Drugs targeting immune checkpoints has been developed and are being extensively tested in preclinical or clinical trials (Daly et al., 2015). Then we enrolled checkpoint members, including PDCD1LG2, PDCD1, LAG3, IDO1, ICOS, HAVCR2, CTLA4, CD274 and C10orf54, into the analysis. And we found that expression of most of these checkpoint genes were significantly associated with risk score (Figure 7E and Supplementary Figure S4E), indicating that the prognostic signature could aid in identification of patients who were more sensitive to immune checkpoint blockade therapies.

### Construction of the B7-CD28 Family-Based Nomogram

**FIGURE 8:**
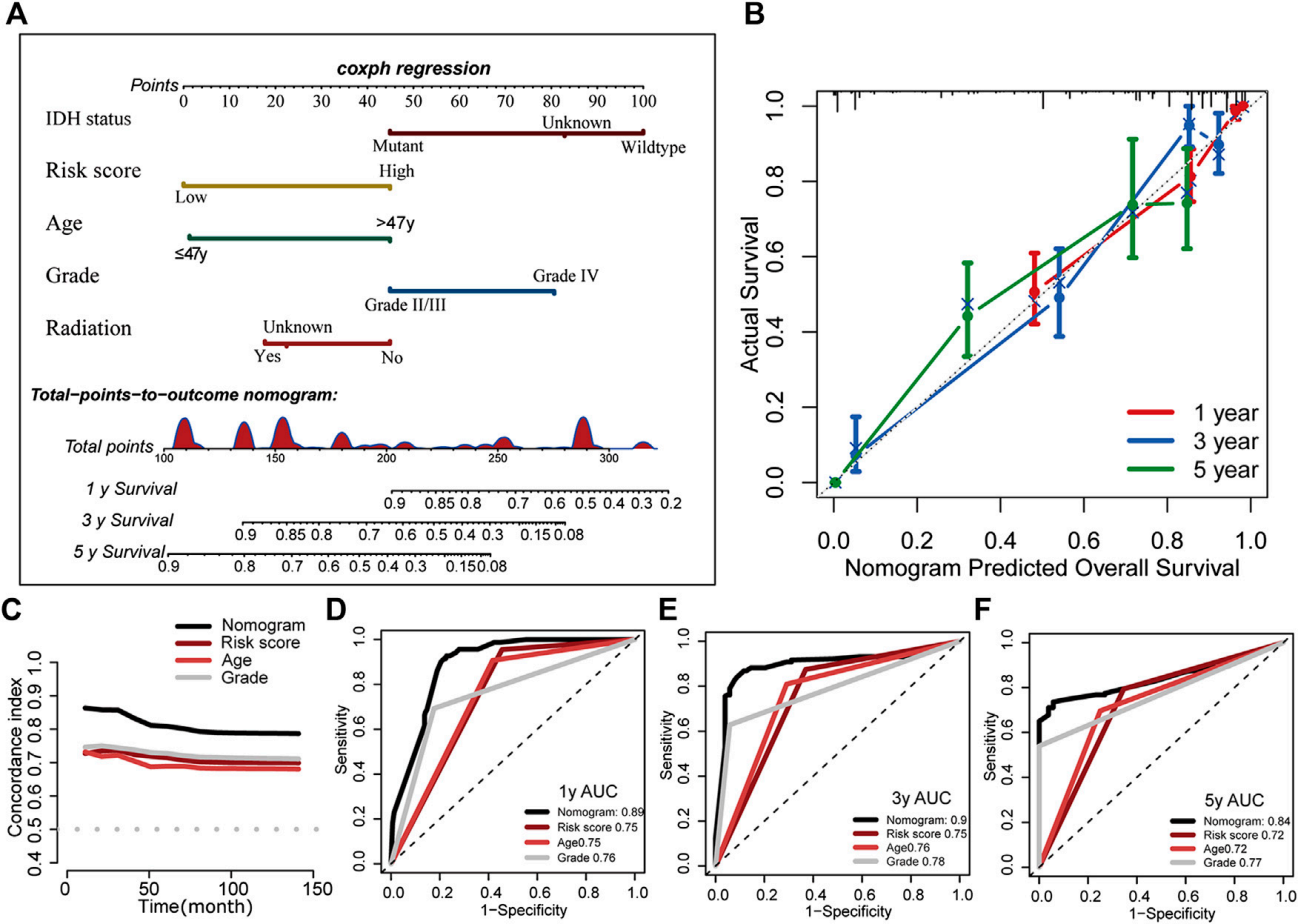
Construction of the B7-CD28 family-based nomogram in TCGA cohort **(A)**. Calibration plot of the nomogram for predicting the probability of overall survival at 1, 3, and 5 years **(B)**. The time-dependent C-index and ROC curve analysis showed that the nomogram had best performance **(C–F)**.

In order to make full use of the gene signature we developed, based on the results of multivariate analysis in TCGA cohort, we established a nomogram in which the signature integrated the other four independent prognostic factors to estimate overall survival for glioma patients (IDH status, age, tumor grade and radiotherapy; Figure 8A). Calibration curves of nomogram-predicted survival vs. actual outcomes demonstrated excellent concordance (Figure 8B). The C-index for the nomogram was 0.852 (95%CI, 0.828–0.877), showing favorable discrimination ability. We also compared the predictive accuracy of this nomogram with individual predictors including gene signature-based risk score, IDH status, tumor grade and patient age. And the time-dependent C-index and ROC curve analysis revealed that the nomogram had best performance, showing that this model was of good stability and powerful prediction ability (Figures 8C–F).

## Discussion

Although considerable advancement has been made in surgical resection, along with radiotherapy and chemotherapy for glioma patients, clinical efficacy of these conventional therapies is still far from satisfactory. Exploring new therapeutic approaches to improve survival for glioma patients is urgently needed in this context. With the rise of immunotherapy, an unprecedented number of clinical trials are under the way to investigate the clinical applicability of immunotherapy in glioma, encouraged by the recent FDA approvals of immune checkpoint inhibitors in serval types of other advanced cancer (Hodges et al., 2017; Jahan et al., 2018). Hence, specific biomarkers that both predict clinical outcome and immunotherapeutic responses while also immunological characteristics in diffuse gliomas are still urgently to be discovered, in an effort to bolster clinical tools for immunotherapeutic response assessment and biological insight of this tumor.

In current study, we systematically explored the association of the B7-CD28 family genes with glioma outcome, and developed a B7-CD28 family-based model significantly related to the survival of glioma patients using TCGA-GBMLGG cohort. We validated its prognostic value in important clinical subgroups and three independent cohorts from CGGA database. In addition, we investigated the role of the gene signature as a predictive immune marker for immunotherapy and tumor microenvironment. Afterwards, we further explored the gene signature-related underlying mechanisms to deepen the understanding of the cross talk between tumor and immune system.

Finally, a B7-CD28-based nomogram was established to predict patient life expectancy contributing to facilitate personalizing therapy for tumor sufferers. Some studies had also used public-access databases with large scale samples to explore the several single B7-CD28 family members in whole grade glioma. Zhang et al. found that CD276 indicated the malignant phenotype of glioma and independently predicted worse prognosis in glioma patients (Zhang et al., 2018). And the association of the CD276 collaborating with other checkpoint members with dysfunctional phenotype of T cell was also observed. However, we conducted a comprehensive analysis on all B7-CD28 family genes in our study to provide in-depth understanding of these genes in gliomas.

The core genes constituted our genetic signature were CD276 (B7-H3), CD274 (PD-L1), PDCD1LG2 (PD-L2) and CD80 (B7-1), belonging to the B7 family. CD276 is overexpressed in various human malignancies, although its receptor has not been identified yet (Zhang et al., 2018). Compared separate B7-CD28 family member, we found CD276 was the strongest factor influencing the outcome. And recent studies have reported that CD276 had functions on T-cell co-inhibition contributing to tumor cell evasion (Wang et al., 2014; Picarda et al., 2016; Lee et al., 2017). Also, CD276 may act as a potent adjunct to facilitating the immune evasion function of macrophages (Zhang et al., 2018) and NK cells. In our study, through specific immune cell population association analysis, the associations of the signature with myeloid dendritic cells and monocytic lineage were also observed, indicating that signature could also act as indicator for the immune evasion function of the immune cells such as macrophages. Better elucidating the involvement of CD276 pathway in immune responses will promote the great development of immunotherapy for glioma. CD274 was commonly expressed on normal cells and immune cells, while PDCD1LG2 mainly expressed on antigen-presenting cells; they bind with programmed cell death protein-1 (PD-1) on T cell surface playing important roles in suppression of T-cell immunity and are, accordingly, important targets for blockade-based immunotherapy in cancer (Yearley et al., 2017; Wang et al., 2019). CD80 (B7-1) and CD86(B7-2) are ligands typically expressed on antigen presenting cells (Zhang et al., 2014). They can interact with CD28 to trigger a costimulatory signal that potentiates T‐cell activation and function, but can also inhibit certain effector T‐cell responses *via* interacting with CTLA-4, contributing to a balance between T‐cell activation and suppression (Stamper et al., 2001; Intlekofer and Thompson, 2013; Chen et al., 2020). And we observed that increased expression of CD80 was a risk factor related to worse survival in our study, suggesting that balance has shifted and interaction between CD80 and CTLA-4 may account for the dominant in diffuse glioma. Thus, targeting CD80 may become an attractive strategy in immunotherapy for glioma. Consistent with all above, genes positively correlated with the signature-based risk score were mainly enriched in immune related process. Through analyzing the association of the signature with inflammatory-related clusters and Treg signatures expression, we uncovered its related biological function involving in inflammation cells transduction signals activation and immunosuppression. Furthermore, the good relationship between the risk score and other immune checkpoints was observed, indicating our prognostic model could also assist in selecting ideal immunotherapies for individual patients and optimizing immunotherapy strategies.

Although the B7-CD28 family gene signature may have substantial clinical value for diffuse glioma, several limitations of this study should be noted as well. Firstly, our study enrolled multi-institutional cohorts for analysis, but for its retrospective nature, further prospective studies are still needed. Meanwhile, the reliability of our molecular results remains challenged without validation *in vitro* or *in vivo* experiments. Secondly, since our model was only in consideration of B7-CD28 family members, it may could reflect more tumor related characteristics but may also lost some prognostic predictive ability. Thirdly, patients we analyzed were not treated by immunotherapy, it is unclear whether the prognostic signature is still stable in patients received such treatment. Finally, our B7-CD28 family-based nomogram could improve predictive performance by incorporating more clinical parameters.

In summary, we identified and validated a B7-CD28 family-based signature that had independent prognostic significance for diffuse glioma patients, and had great potential to reflect the clinicopathologic, molecular, and immunological features of the tumor. Our workflow was summarized in Supplementary Figure S5. Considering the crucial role of B7-CD28 family in development of immunotherapy, underlying mechanisms should be further elucidated to find out more ideal therapeutic targets. Moreover, this is the first mathematical model based on this gene family with the aim of providing novel insights into immunotherapy for diffuse glioma, and further validations were also required.

## Data Availability

The datasets presented in this study can be found in online repositories. The names of the repository/repositories and accession number(s) can be found in the article/Supplementary Material.
