# Supplementary figures and images for: A B7-CD28 Family-Based Signature Demonstrates Significantly Different Prognosis and Immunological Characteristics in Diffuse Gliomas
Source: Front Mol Biosci. 2022 Jul 19;9:849723. doi: 10.3389/fmolb.2022.849723 (PMC9344576; doi:10.3389/fmolb.2022.849723)

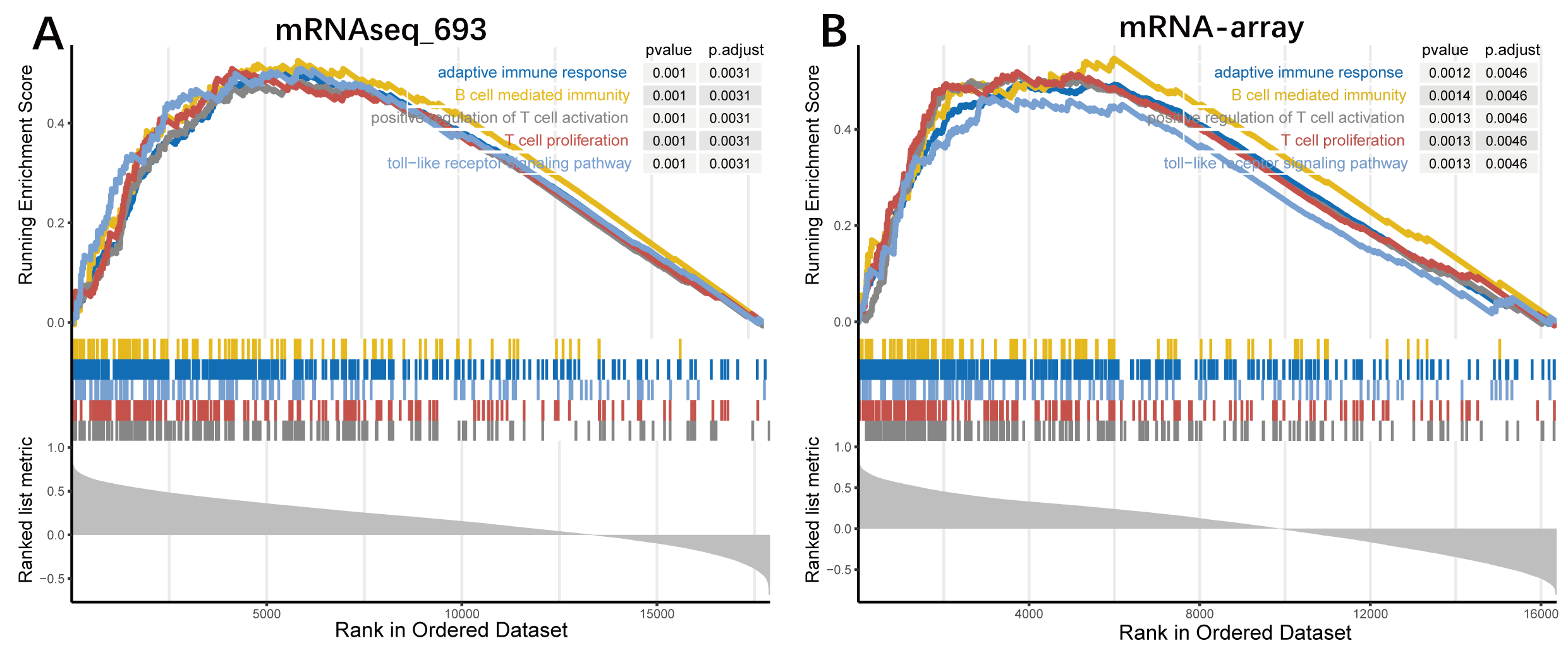

Supplement: Supplementary file 2 [file Image3.TIF]

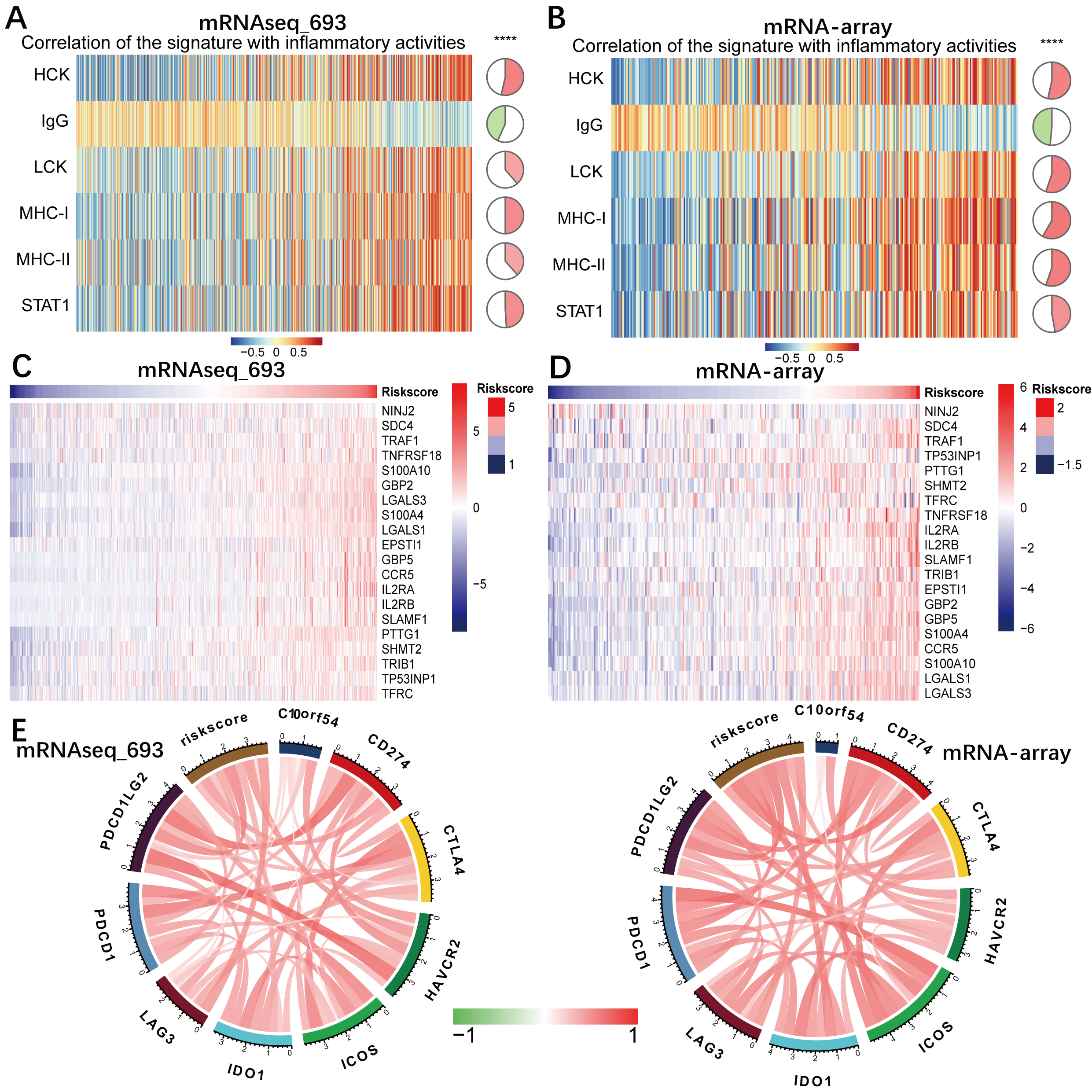

Supplement: Supplementary file 3 [file Image4.TIF]

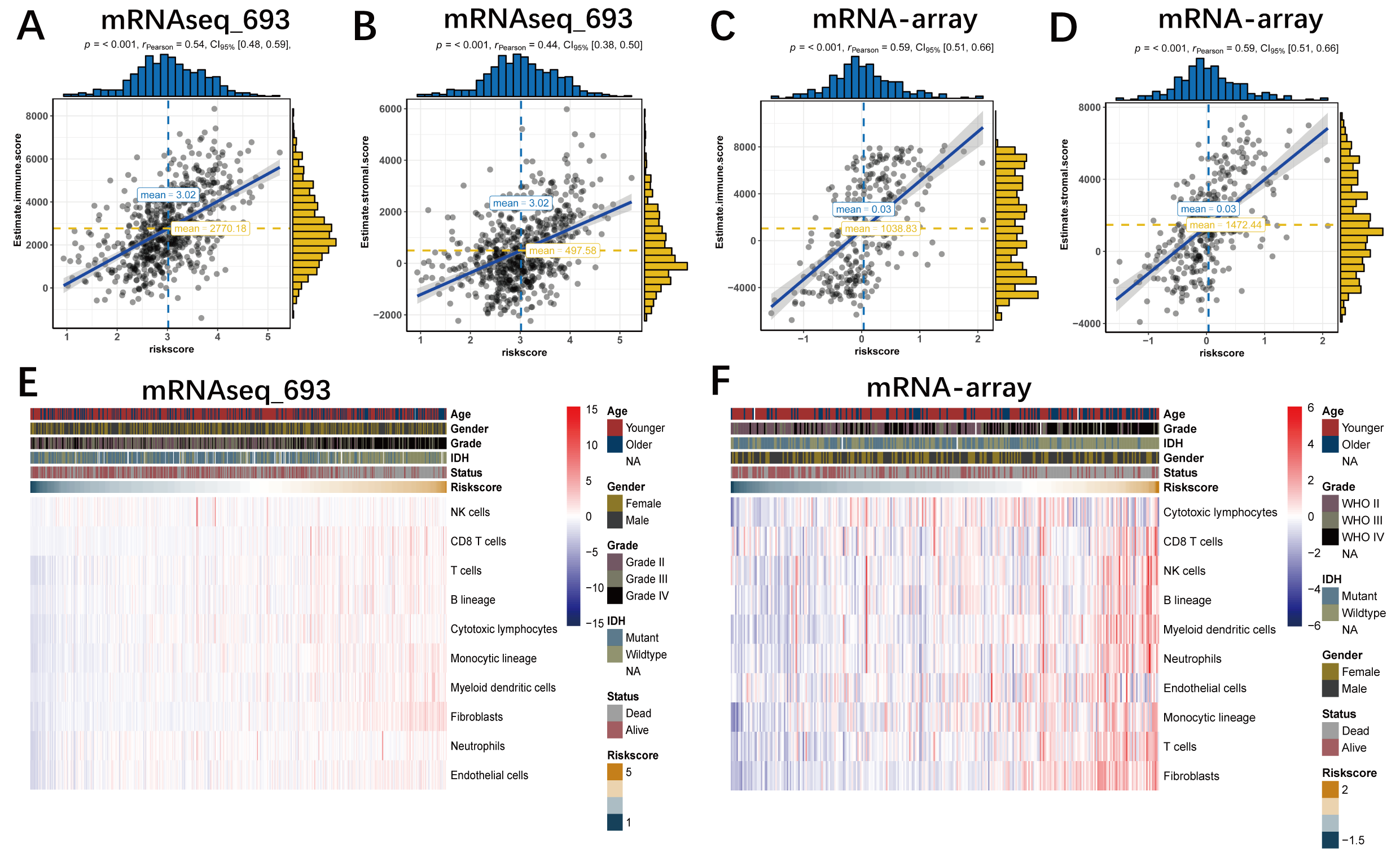

Supplement: Supplementary file 4 [file Image2.TIF]

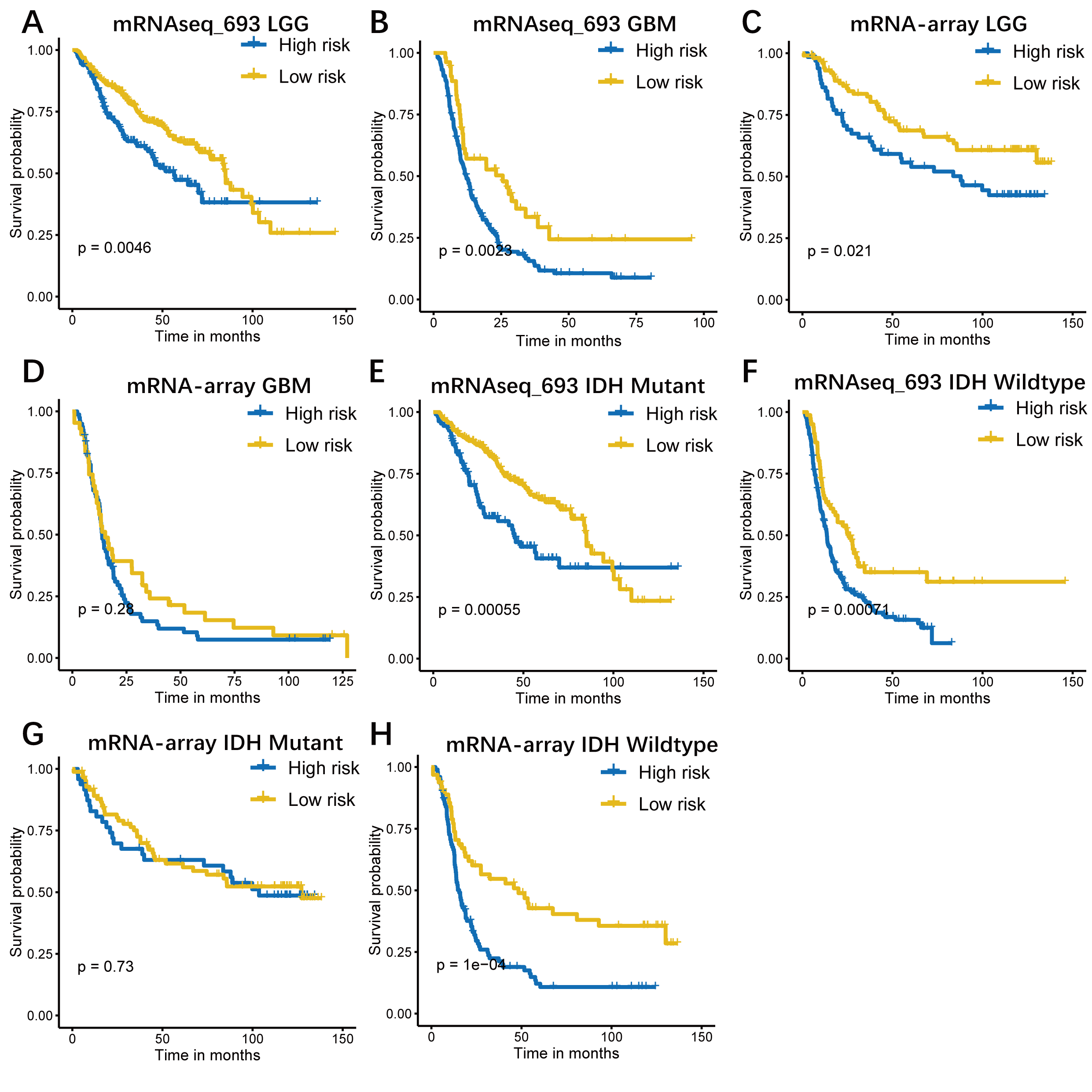

Supplement: Supplementary file 5 [file Image1.TIF]

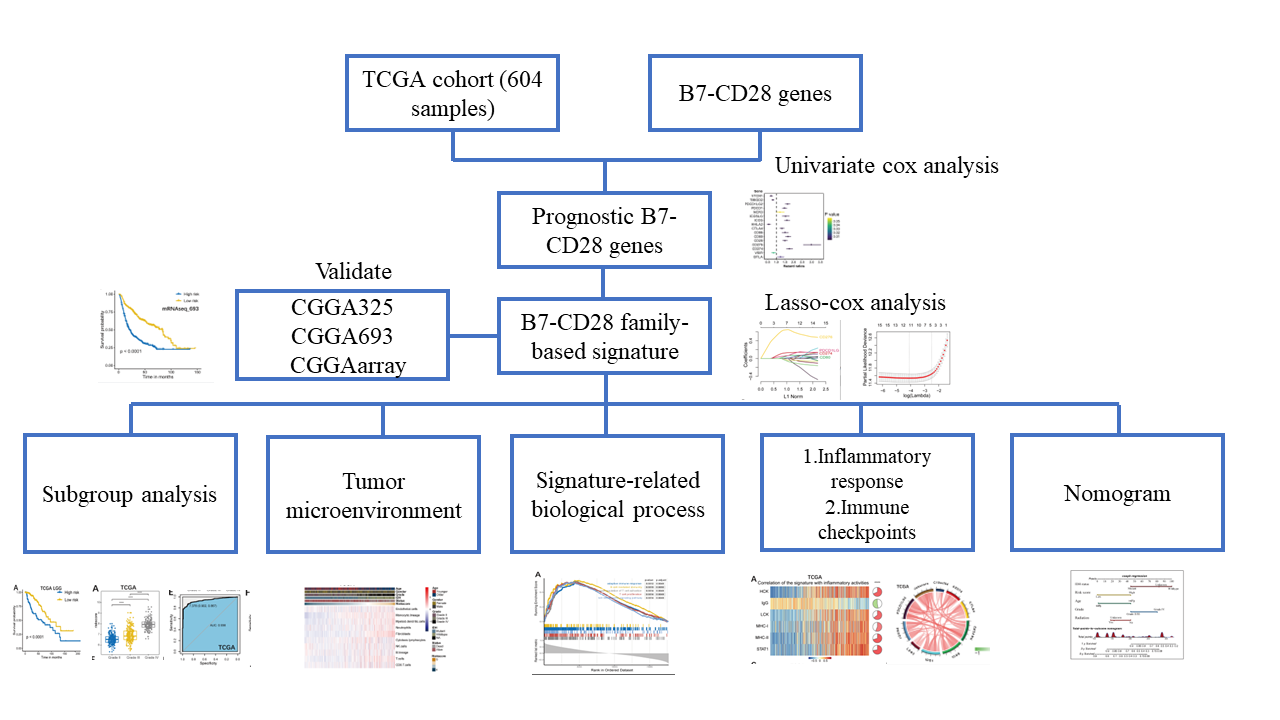

Supplement: Supplementary file 7 [file Image5.TIF]
